# Supplementary material for: Bleaching correction for DNA measurements in highly diluted solutions using confocal microscopy
Source: PLoS One. 2020 Jul 23;15(7):e0231918. doi: 10.1371/journal.pone.0231918 (PMC7377397; doi:10.1371/journal.pone.0231918)
Supplement: S1 Appendix — (PDF) [file pone.0231918.s001.pdf]

Supporting information

Linear model. In addition to the detailed derivation of the rotation for a second-order polynomial, we show the less complex model for the rotation of a straight line here. We start with the straight line equation

$$I = f(C) = a\,C + const. \tag{21}$$

A rotation matrix is applied to the straight line.

$$\vec{r'} = \begin{pmatrix} C' \\ I' \end{pmatrix} = R_\theta \vec{r} = \begin{pmatrix} \cos\theta & -\sin\theta \\ \sin\theta & \cos\theta \end{pmatrix} \begin{pmatrix} C \\ I \end{pmatrix} \tag{22}$$

If the Y-axis intersection is omitted, the following expression is obtained after multiplication.

$$C'(\theta) = C\cos\theta - I\sin\theta = C\cos\theta - (a\,C)\sin\theta \tag{23}$$

$$I'(\theta) = C\sin\theta + I\cos\theta = C\sin\theta + (a\,C)\cos\theta \tag{24}$$

Eq 23 is reshaped to C.

$$C(\theta) = \begin{cases} \frac{C'}{\cos\theta - a\sin\theta}, & \text{for } [\theta \neq 0] \\ C', & \text{for } [\theta = 0] \end{cases} \tag{25}$$

Eq 25 is now inserted in Eq 24 and the y intersection *const* is considered.

$$I'(C') = \frac{C'\sin\theta}{\cos\theta - a\sin\theta} + \frac{C' a\cos\theta}{\cos\theta - a\sin\theta} + const \tag{26}$$

For the reverse calculation from the measured intensity I' from the experiment to the actual concentration, Eq 26 is reshaped to C'.

$$C'(\theta) = \frac{(I' - const)(\cos\theta - a\sin\theta)}{\sin\theta + a\cos\theta} \tag{27}$$

In the case of smaller concentration ranges, where a linear model describes the dilution series with sufficient accuracy, this less complex relationship can be used to calculate the corrected mass concentration.

Table S1 Artificial mixtures. Mass determination of eleven mixtures with four defined mass concentrations (20 pg/µl, 50 pg/µl, 100 pg/µl and 200 pg/µl)

Mixture	20 pg/µl	50 pg/µl	100 pg/µl	200 pg/µl
50 bp:1000 bp (1:1)	21.0	47.8	111.4	214.2
50 bp:1000 bp (1:2)	17.5	52.0	97.6	177.0
50 bp:1000 bp (1:3)	20.5	49.3	92.9	210.9
50 bp:1000 bp (1:4)	19.0	49.7	93.8	208.3
50 bp:1000 bp (1:5)	19.6	44.8	96.7	190.5
50 bp:1000 bp (1:6)	17.5	45.4	104.1	165.7
200 bp:500 bp (1:1)	25.0	55.0	99.6	204.9
200 bp:500 bp (1:2)	23.8	53.0	93.7	206.7
200 bp:500 bp (1:3)	16.1	42.7	94.5	200.7
200 bp:500 bp (1:4)	30.5	51.4	95.8	202.5
200 bp:500 bp (1:5)	28.4	46.7	104.0	195.6
Mean [pg/µl]	21.7	48.9	98.5	197.9
Variance coeffizient [%]	23.3	7.60	5.8	7.5
Deviation from target[%]	8.6	-2.2	-1.5	-1.1

Table S2 Natural mixtures. Mass determination of eight NGS libraries with defined mass concentrations (10 pg/µl, 20 pg/µl and 50 pg/µl) diluted from 1000 pg/µl

Mixture	10 pg/µl	20 pg/µl	50 pg/µl
Library 1	8.6	22.0	49.8
Library 2	7.5	18.8	42.8
Library 3	9.2	15.0	41.3
Library 4	12.0	19.1	53.1
Library 5	9.3	25.5	56.2
Library 6	9.8	22.0	52.4
Library 7	9.9	17.9	46.7
Library 8	9.8	17.8	46.4
Mean [pg/µl]	9.51	19.8	48.6
Variance coeffizient [%]	12.9	16.3	10.4
Deviation from target[%]	-4.7	-1.2	-2.8
